# Pro-Oxidant Activity of Amine-Pyridine-Based Iron Complexes Efficiently Kills Cancer and Cancer Stem-Like Cells

**DOI:** 10.1371/journal.pone.0137800

**Published:** 2015-09-14

**Authors:** Marta González-Bártulos, Clara Aceves-Luquero, Jamal Qualai, Olaf Cussó, Mª Angeles Martínez, Silvia Fernández de Mattos, Javier A. Menéndez, Priam Villalonga, Miquel Costas, Xavi Ribas, Anna Massaguer

**Affiliations:** 1 Department of Biology, University of Girona, Girona, Catalunya, Spain; 2 Departament de Biologia Fonamental and Institut Universitari d’Investigació en Ciències de la Salut (IUNICS), Universitat de les Illes Balears, Illes Balears, Spain; 3 Department of Chemistry, University of Girona, Girona, Catalunya, Spain; 4 Institut de Química Computacional i Catàlisi (IQCC), University of Girona, Girona, Catalunya, Spain; 5 Translational Research Laboratory, Catalan Institute of Oncology (ICO), Girona, Catalunya, Spain; 6 Girona Biomedical Research Institute (IDIBGI), Girona, Catalunya, Spain; National Institute of technology Rourkela, INDIA

## Abstract

Differential redox homeostasis in normal and malignant cells suggests that pro-oxidant-induced upregulation of cellular reactive oxygen species (ROS) should selectively target cancer cells without compromising the viability of untransformed cells. Consequently, a pro-oxidant deviation well-tolerated by nonmalignant cells might rapidly reach a cell-death threshold in malignant cells already at a high setpoint of constitutive oxidative stress. To test this hypothesis, we took advantage of a selected number of amine-pyridine-based Fe(II) complexes that operate as efficient and robust oxidation catalysts of organic substrates upon reaction with peroxides. Five of these Fe(II)-complexes and the corresponding aminopyridine ligands were selected to evaluate their anticancer properties. We found that the iron complexes failed to display any relevant activity, while the corresponding ligands exhibited significant antiproliferative activity. Among the ligands, none of which were hemolytic, compounds **1**, **2** and **5** were cytotoxic in the low micromolar range against a panel of molecularly diverse human cancer cell lines. Importantly, the cytotoxic activity profile of some compounds remained unaltered in epithelial-to-mesenchymal (EMT)-induced stable populations of cancer stem-like cells, which acquired resistance to the well-known ROS inducer doxorubicin. Compounds **1**, **2** and **5** inhibited the clonogenicity of cancer cells and induced apoptotic cell death accompanied by caspase 3/7 activation. Flow cytometry analyses indicated that ligands were strong inducers of oxidative stress, leading to a 7-fold increase in intracellular ROS levels. ROS induction was associated with their ability to bind intracellular iron and generate active coordination complexes inside of cells. In contrast, extracellular complexation of iron inhibited the activity of the ligands. Iron complexes showed a high proficiency to cleave DNA through oxidative-dependent mechanisms, suggesting a likely mechanism of cytotoxicity. In summary, we report that, upon chelation of intracellular iron, the pro-oxidant activity of amine-pyrimidine-based iron complexes efficiently kills cancer and cancer stem-like cells, thus providing functional evidence for an efficient family of redox-directed anti-cancer metallodrugs.

## Introduction

Cancer cells undergo metabolic adaptations to sustain their uncontrolled growth and proliferation. Diverse intrinsic and extrinsic molecular mechanisms contribute to this metabolic reprogramming to supply cancer cells with sufficient energy and biosynthetic capacity in the tumor environment [1,2]. Altered metabolism together with activated oncogenic signaling and deregulation of mitochondrial function typically results in an increase in the generation of reactive oxygen species (ROS) in cancer cells [3,4]. Interestingly, this phenomenon leads to a differential redox homeostasis in normal and malignant cells that is gaining ground as a promising target for the design of more selective and effective anticancer agents [5–8].

Highly reactive ROS are produced in cells by the incomplete reduction of molecular oxygen to water during aerobic metabolism. ROS are normally regulated by cellular defensive antioxidants [9,10] and participate in multiple cellular functions including signal transduction, enzyme activation, gene expression and protein post-translational modifications [11]. When generated in excess or when the efficiency of the cellular antioxidant system is submaximal, ROS accumulate and cause irreversible cellular damage through the oxidation of biomolecules such as lipid membranes, enzymes or DNA which generally leads to cellular death [12]. ROS can also promote cancer initiation and progression by inducing DNA mutations and pro-oncogenic signaling pathways [13,14].

Increased ROS in cancer cells upregulates the antioxidant response, resulting in a new redox balance that enables these cells to maintain higher ROS levels than normal cells. Consequently, cancer cells exhibit persistent oxidative stress, which promotes cell proliferation but is insufficient to cause cellular death [4,13]. This altered homeostasis renders cancer cells vulnerable to exogenous oxidizing agents that generate additional ROS, which are likely to increase oxidative stress levels above the cytotoxic threshold. This susceptibility is heightened by the restricted capacity of cancer cells to strengthen the antioxidant response to neutralize the oxidative insult [15]. In contrast, normal cells can tolerate higher levels of exogenous ROS stress since they exhibit lower constitutive ROS levels together with a superior responsiveness of antioxidant systems. In fact, it is well described that, in addition to their direct effects on DNA and cell division, the mechanism of action of many chemotherapeutic agents such as 5-fluoruracil, bleomycin, cisplatin, doxorubicin or paclitaxel also involves ROS-mediated apoptosis [13,16–19].

While the biological effects of ROS and the mechanisms regulating ROS levels are well established in cancer cells, little is known about the role of ROS in the cancer stem cell (CSC) subpopulation, which displays a high capacity for self-renewal and differentiation and also the potential to generate tumors with a marked chemo-/radio resistance [20,21]. CSCs contain lower levels of ROS than non-CSCs, likely as a consequence of enhanced free radical scavenging systems [22]. Low ROS levels might be related to the privileged status of this subset of cells, preserving DNA integrity and protein function, which is critical to maintain the potential for self-renewal and stemness [23,24]. Thus, exogenous ROS elevation might be an approach to kill the CSC subpopulation, which is normally enriched after conventional chemotherapy. Indeed, niclosamide and arsenic trioxide (AS_2_O_3_), which are potent ROS inductors, have been shown to promote CSC death [25].

A number of anticancer agents that target the cellular redox balance are in different phases of preclinical and clinical development [5,6]. Mechanistically, these agents either inhibit the cellular antioxidant defense systems [27–29] or generate ROS [30–32]. In addition to these agents, transition metal-based compounds may be promising candidates for pro-oxidant therapies. When accumulated in cells, metals such as iron, manganese and copper, undergo cycling redox reactions that generate high levels of ROS, principally the highly-damaging hydroxyl radical species through the Fenton reaction. This metal-mediated form of oxidative stress is a well-known cause of cell death [33], and thus, an increasing number of investigations are exploring the potential of metallodrugs in redox-based anticancer therapies [34–37].

Transition metal complexes with aminopyridine-containing organic scaffolds have emerged as powerful catalysts for the oxidation of organic substrates. These complexes are also regarded as bioinspired catalysts since they reproduce structural and reactivity properties of oxidative enzymes. A key aspect of their activity is their strong binding to iron and manganese ions, generating powerful oxidants after reacting with peroxides [38–43]. These oxidant compounds function as catalysts to promote the oxidation of inert molecules such as alkanes, alkenes and even the challenging water molecule. The mechanism of action involves ferric-peroxide species, chemically reminiscent to activated bleomycin. In addition, these compounds are highly resistant to self-oxidation. With this background, we here assessed the antiproliferative and cytotoxic activity profiles of five amino-pyridine-based Fe(II)-complexes which have been previously shown to be particularly active in peroxide activation reactions [38–43], and the corresponding metal-free ligands, against a panel of diverse human cell lines including epithelial-to-mesenchymal (EMT)-induced stable populations of cancer stem-like cells and non-malignant cells. The most active compounds were further analyzed for their ability to inhibit the clonogenicity of cancer cells, modulate the cell cycle and induce cell death. The capacity of the amine-pyrimidine-based iron complexes to generate ROS and cause DNA damage was evaluated together with the influence of the chelation of intracellular iron on their cytotoxic profile. Based on the lethal disruption in the redox balance caused by these complexes in cancer and ROS-resistant cancer stem-like cells, we provide strong functional evidence for an efficient family of redox-directed anti-cancer metallodrugs.

## Materials and Methods

### Materials

3-(4,5-Dimethylthiazol-2-yl)-2,5-diphenyltetrazolium bromide (MTT), dimethyl sulfoxide (DMSO), propidium iodide (PI), deferoxaminemesylate salt (DFO), N-acetyl-L-cysteine (NAC), calcein-acetoxymethylester (calcein-AM), cacodylate buffer, Tris-EDTA (ethylene-diamino tetracetic acid), tiron, sodium azide, methyl green, Hoechst, ethidium bromide, bromophenol blue, xylene cyanol, glycerol and RNase A were from Sigma Aldrich (St. Louis, MO, USA). Methylene blue, hydrogen peroxide 35% (w/v) and ethanol were from Panreac (Barcelona, Spain). HEPES was from ICN (Madrid, Spain). Triton-X100 was from PlusOne (Amersham Bioscience, Uppsala, Sweden). 2′,7′-Dichlorodihydrofluorescein diacetate (H_2_DCFDA) was from Molecular Probes (Invitrogen, Life Technologies, Carlsbad, CA, USA). Agarose was from Ecogen (Barcelona, Spain). Cisplatin (Pharmacia, Pfizer Inc, Kalamazoo, MI, USA) was kindly provided by the pharmacy of the Catalan Institute of Oncology (ICO, Hospital Dr. Josep Trueta, Girona, Spain). Dulbecco’s modified Eagle’s medium (DMEM), RPMI-1640 medium, phosphate buffered saline (PBS), fetal bovine serum (FBS), penicillin-streptomycin and trypsin were obtained from GIBCO BRL (Grand Island, NY, USA). DMEM/F12, horse serum and insulin were from Invitrogen. Hydrocortisone, cholera toxin and epidermal growth factor were from Sigma-Aldrich. Mammary Epithelial Cell Growth Medium (MEGM) was from Lonza (Berkshire, UK). The pUC18 plasmid was from Thermo Scientific (Waltham, MA, USA). Compounds selected for this study (**1, 1-Fe, 2, 2-Fe, 3, 3-Fe, 4, 4-Fe, 5 and 5-Fe**) were synthesized following reported procedures [38–41].

### Cell lines

Human MCF-7 breast cancer cell line, CAPAN-1 pancreatic cancer cell line, PC-3 prostate cancer cell line, Z-138, Jeko-1, Granta and SP53 non-Hodgkin's lymphoma cell lines, JURKAT T-cell acute lymphoblastic leukemia cells, LN229 and U87MG glioma cell lines and MCF 10A immortalized mammary epithelial cell line were obtained from the American Type Culture Collection (ATCC, Rockville, MD, USA). The CCD-18Co human colon fibroblast cell line was obtained from EucellBank (University of Barcelona, Barcelona, Spain). 1BR3G transformed human skin fibroblasts were obtained from the European Collection of Cell Cultures (ECACC, Porton, UK). MCF-7, CAPAN-1, PC-3, LN229 and U87MG were maintained in DMEM. Hematological cell lines were maintained in RPMI-1640. All media were supplemented with 10% FBS and 100 U/ml penicillin-streptomycin. Medium for JURKAT, Jeko-1 and Z-138 cells was supplemented with 25 mmol/L HEPES. MCF 10A cells were maintained in DMEM/F12 supplemented with 5% horse serum, 500ng/ml hydrocortisone, 100ng/ml cholera toxin, 10 μg/ml insulin and 20ng/ml epidermal growth factor. HMLE cells (immortalized human mammary epithelial cells), HMLER cells (HMLE cells overexpressing hTERT, SV40 T/t and H-RasV12) and HMLERshEcad cancer stem-like cells (HMLER cells transformed via short hairpin RNA to inhibit expression of the CDH1 gene, which encodes for E-cadherin) [44], were maintained in a 1:1 mixture of HMLE medium (DMEM/F-12 plus 5% horse serum, penicillin-streptomycin-glutamine (PSG), 10 μg/mL insulin, 10 ng/mL epidermal growth factor and 0.5 g/mL hydrocortisone) and MEGM. All cell lines were grown at 37°C under a humidified atmosphere containing 5% CO_2_

### Cytotoxicity Assays

The cytotoxic activity of the compounds was determined by MTT reduction assay as described [45]. Compounds were diluted in Milli-Q water to obtain 1 mmol/L stock solutions. Appropriate aliquots of these solutions were diluted in the corresponding cell culture medium to obtain the final working concentrations. Aliquots of 5000 1BR3G cells, 6000 MCF-7, 6000 PC-3 cells, 10 000 CAPAN-1 cells, 4000 MCF 10A cells, 4000 HMLE cells or 4000 CCD-18Co cells were seeded in 96-well plates, 24 h prior to the treatments. Hematological cell lines were seeded at 400 000 cells/mL. Cells were treated with the corresponding compound at concentrations ranging from 0 to 100 μmol/L for 48 h. Three replicates for each compound were used. The IC_50_ was established for each compound by standard non-linear regression and curve fitting using GraphPad Prism (Graph Pad software Inc., La Jolla, CA, USA).

### Hemolytic assay

The hemolytic activity of the compounds at 100 μmol/L was evaluated by determining hemoglobin release from erythrocyte suspensions of fresh human blood (5% vol/vol) as described [46].

### Colony formation assay

MCF-7 cells were seeded in 12-well plates. Twenty-four-hours later, cells were treated with cisplatin, compound **1**, **2** or **5** at 10 μmol/L, or vehicle alone as a control, for 3 and 24 h at 37°C. Additionally, cells were exposed to compound **1** for 3, 6, 12 and 24 hours. Subsequently, cells were washed with PBS, collected with trypsin and plated at low density (3000 cells in a 360-mm plate). Cells were allowed to divide and form colonies for 7–10 days; after which, colonies were fixed and stained with 2% methylene blue in 50% ethanol. The number of colonies in each plate was determined using the Alpha Innotech Imaging system (Alpha Innotech, San Leandro, CA).

### Caspase activity analysis

Enzymatic caspase activity was determined after exposing the cells to compound **1**, **2** and **5** at 10 μmol/L for 48 h. Caspase 3/7 activity was measured with the luminometric Caspase-Glo 3/7assay (Promega, Madison, WI, USA) using a Synergy HT multi-detection microplate reader (Bio-Tek).

### Cell cycle analysis

Cell cycle profiles were analyzed by flow cytometry of PI-stained cells. Briefly, cells were collected by centrifugation, washed in ice-cold PBS and fixed for 30 min at 4°C in 70% ethanol. After washing twice with PBS, DNA was stained with 50 μg/mL PI in the presence of 50 μg/ml RNase A. Stained cells were then processed using a FACScan flow cytometer (Coulter Epics XL-MSL; Beckman Coulter, Fullerton, CA, USA) and winMDI software.

### ROS mesurement

Cellular ROS content was determined using the 2′,7′-dichlorodihydrofluorescein diacetate probe (H_2_DCFDA). Cells were seeded in 24-well plates (50 000 cells/well) in phenol red-free DMEM 24 h prior to treatments. Cells were treated with different concentrations of compound **1**, **2** and **5** (2.5, 5 or 10 μmol/L) or vehicle alone as a control, for 5 or 24 hours at 37°C. In some experiments, cells were co-treated with the compounds plus 5 mmol/L NAC. After treatments, cells were washed with PBS and incubated with 1 μmol/L H_2_DCF-DA in PBS for 30 minutes in the dark. After washing, cells were collected with trypsin and analyzed by flow cytometry using a FACS-Calibur flow cytometer (Becton-Dickinson®, Immunofluorometry Systems, Mountain View, CA, USA). The geometric mean fluorescence intensity of 10 000 cells was established using CellQuestTM software (Becton Dickinson). The fluorescence fold-increase versus untreated cells was determined for each treatment.

### Determination of cellular labile iron pool

The cellular labile iron pool was determined with calcein-AM. CAPAN-1 cells (125 000 cells/ well) were seeded in 24-well plates and incubated for 24 h. Then, cells were treated for 24 h with 10 μmol/L of compound **1**, **2** or **5** at 37°C. In some experiments, cells were incubated for 2 h with 100 μmol/L of DFO or 100 μmol/L FeCl_2_. Cells exposed to the vehicle alone were used as a control. After treatments, cells were washed with PBS and incubated with calcein-AM (0.25 μmol/L) for 30 min at 37°C in the dark. Subsequently, cells were washed and collected with trypsin and the geometric mean fluorescence intensity of 10 000 cells was determined by flow cytometry as described.

### Cellular DNA damage analysis

DNA damage was assessed by monitoring the intensity of p-H2A.X fluorescence using flow cytometry. Briefly, cells were collected with trypsin, washed in PBS and fixed in 3.7% formaldehyde for 15 min on ice. Cells were then permeabilised with 0.2% v/v Triton-X100 for 10 min and incubated with 1:400 rabbit anti-p-(S139)-H2A.X antibody (Cell Signaling Technology, Danvers, MA) for 30 min on ice. After washing in 0.1% Triton-X100 in PBS, cells were incubated with 1:400 anti-rabbit Alexa 555-conjugated antibody (Jackson ImmunoResearch, Newmarket, UK) for 20 min on ice. Analysis was carried out in a FACScan flow cytometer with Flowing software.

### DNA cleavage analysis

DNA cleavage was monitored by agarose gel electrophoresis. A stock solution of pUC18 DNA was freshly prepared in Milli-Q water at a concentration of 0.5 μg/mL (1512 μmol/L nucleotides; 756 μmol/L bp). Reactions were performed by mixing 0.5 μl of pUC18 with appropriate aliquots of the compounds and 1 μL of activating agent solution (35% wt/vol H_2_O_2_ in H_2_O). Cacodylate buffer (0.1 M, pH 6.0) was added to the mixture to give a final volume of 20 μl. The final concentration of pUC18 DNA was 37.8 μmol/L in nucleotides (18.9 μmol/L bp). Samples were incubated for 1 h at 37°C; reactions were quenched by adding 6 μL of a buffer solution consisting of bromophenol blue (0.25%), xylene cyanol (0.25%), and glycerol (30%). Subsequently, the samples were subjected to electrophoresis in 0.8% agarose gels in 0.5×TBE buffer (0.045 mol/L Tris, 0.045 mol/L boric acid, and 1 mmol/L EDTA) at 100 V for 1 h and 40 min. Gels were stained with ethidium bromide (10 mg/mL in TBE) for 15 min and visualized under UV transillumination. DNA bands were captured using the ProgRes CapturePro 2.7 system and the intensity of each band was quantified with the GelQuant version 2.7 software (DNR Bio-Imaging Systems, Jerusalem, Israel) using a correction factor of 1.31 to compensate for the reduced ethidium bromide uptake of supercoiled plasmid pUC18 DNA [47]. The proportion of different forms of plasmid DNA was established for each treatment. To test the involvement of ROS in strand scission and possible complex–DNA interaction sites, various ROS scavengers and groove binders were added to the reaction mixtures. The scavengers used were Tiron (10 mmol/L), sodium azide (0.4 mol/L), and dimethyl sulfoxide (DMSO, 3 μL). The groove binders used were methyl green (20 mmol/L) and Hoechst (40 μmol/L).

### Statistical analysis

Statistical analysis was performed with SPSS statistical software for Windows (version 15.0; SPSS Inc., Chicago, IL, USA). Quantitative variables were expressed as mean and standard deviation (SD) of at least three independent experiments. The normality of the data was tested using the Shapiro-Wilk test. The differences between data with normal distribution and homogeneous variances were analyzed using the parametric Student’s t test. A value of p<0.05 was considered significant.

## Results

### Selection of compounds

**Fig 1:**
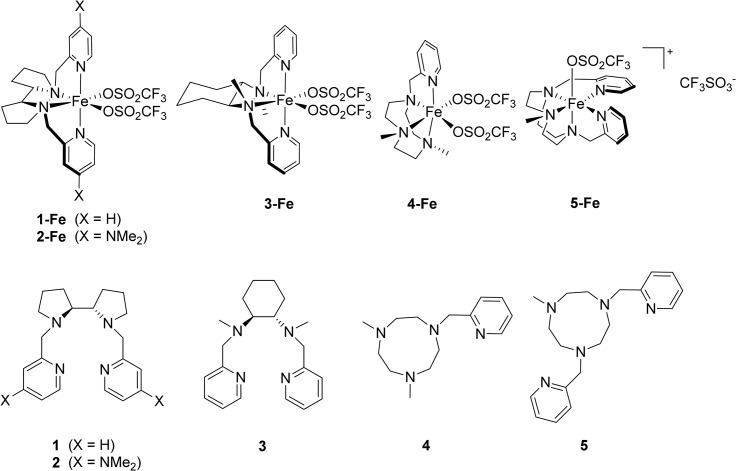
Structures of the iron complexes and the corresponding iron-free organic compounds.

Iron coordination complexes **1-Fe, 2-Fe, 3-Fe, 4-Fe and 5-Fe** were selected to assay their antitumor activity against different human cell lines based on their ability to form highly oxidizing species when reacting with peroxides [38–43]. Four Fe(II)-based complexes **1-Fe, 2-Fe, 3-Fe and 4-Fe** contain tetradentate aminopyridine ligands and are known for their superior activity in oxidation catalysis [38–40,42,43]. Complex **5-Fe** contains a pentadentate ligand and is known to stabilize high oxidation states of iron [41]. Additionally, the iron-free organic compounds **1**, **2**, **3**, **4** and **5** were tested to evaluate the effect of metal ligation for their cytotoxic activity (Fig 1).

### Compounds 1, 2 and 5 are highly cytotoxic against cancer and cancer stem-like cells

**Fig 2:**
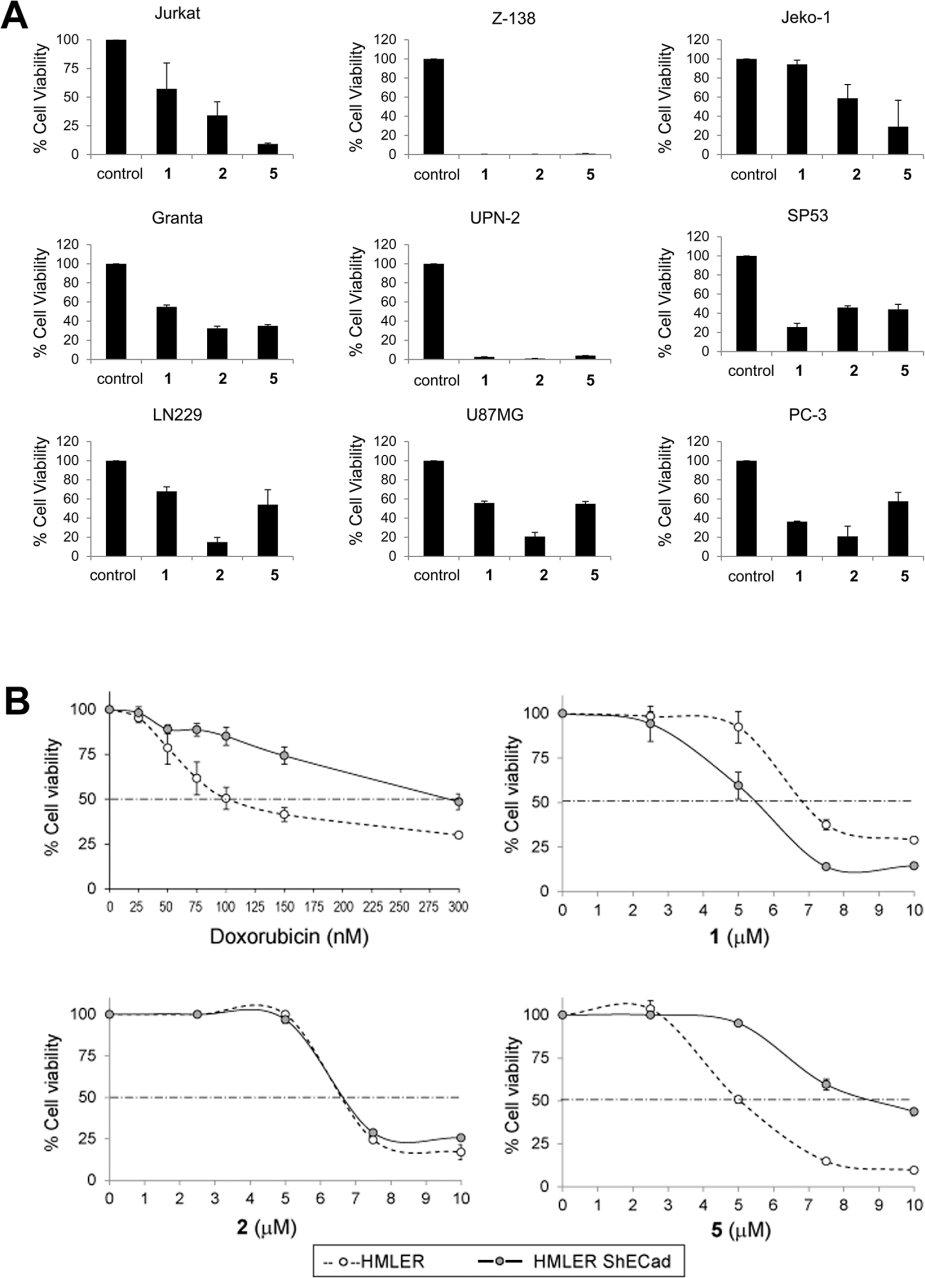
Cell viability assay. (A) Cytotoxic activity of compounds **1**, **2**, and **5** against cancer cells. The indicated cell lines were treated for 48 h with ligands (10 μmol/L) and cell viability was measured with the MTT assay. Data represents the percentage of viable cells relative to untreated cells (control). (B) Cytotoxicity of doxorubicin and compounds **1**, **2** and **5** against CS-like cells. CS-like HMLER-shEcad cells and non-CS-like HMLER isogenic parental cells were treated for 48 h with graded concentrations of doxorubicin, **1**, **2** and **5**. Cell viability was measured with the MTT assay. For each treatment, the percentage of viable cells relative to untreated cells is indicated. Data represents the mean±SD of 3 independent experiments performed in triplicate.

Antiproliferative activity of the iron complexes (**1-Fe, 2-Fe, 3-Fe, 4-Fe and 5-Fe**) and the corresponding iron-free ligands (**1**, **2**, **3**, **4** and **5**) was initially tested against two tumor cancer cells lines, MCF-7 and CAPAN-1. Compounds were tested at different concentrations ranging from 0 to 100 μmol/L to determine the concentration required to inhibit cell growth by 50% (IC_50_). Compounds with IC_50_ values greater than 100 μmol/L were considered to be inactive. Only three of the five iron complexes (**1-Fe, 3-Fe, 4-Fe**) demonstrated a measurable antiproliferative effect in MCF-7 cells, while none of iron complexes were active against CAPAN-1 cells (Table 1). The antiproliferative activity of **3-Fe** and **4-Fe** was rather modest (IC_50_ = 73.5±0.7 μmol/L and 63.5±2.1 μmol/L, respectively). In contrast, all iron-free ligands were cytotoxic in both cell lines analyzed, with IC_50_ values ranging from 3.7±0.4 to 88.5±0.7 μmol/L in MCF-7 cells and from 6.0±0.7 to 32.0±10.4 μmol/L in CAPAN-1 cells. These values are within the range of well-established anticancer agents such as cisplatin assayed under the same conditions (Table 1). Given the weak antiproliferative activity of the iron complexes, we focused on the metal-free organic compounds and evaluated their cytotoxicity against a selection of tumor (PC-3, Z-138 and JURKAT) and non-malignant (HMLE, MCF 10A, 1BR3G and CCD-18Co) cell lines (Table 2). Compound **2** was the most active ligand against tumor cells, with IC_50_ values ranging from 3.8±0.2 to 7.2±1.9 μmol/L. Compounds **1** and **5** also exhibited low IC_50_ values (from 4.8±1.2 to 15.1±3.1 μmol/L and from 2.9±0.4 to 7.7±0.3 μmol/L, respectively), while a more moderate antitumor activity was obtained for ligands **3** and **4**. Only in the normal colon CCD-18Co cell line the activity of the compounds was lower than in tumor cells lines. Importantly, none of the ligands were hemolytic, even at 100 μmol/L (Table 2). The antitumor properties of the ligands were further evaluated in a panel of cell lines, including human leukemia, lymphoma and glioma cancer cells, by analyzing their cytotoxic effects at 10 μmol/L. As anticipated, compounds **1**, **2** and **5** also displayed high antiproliferative activity against these cell lines (Fig 2A), demonstrating their ability to be broadly active antitumor agents.

**Table 1:** Cytotoxicity of the iron complexes and the corresponding ligands against MCF-7 and CAPAN-1 cells.

IC_50_ (μM) ^*a*^		
	Cell line	
Compound	MCF-7	CAPAN-1
**1-Fe**	17.5 ± 0.9	>100
**1**	7.4 ± 0.2	9.9 ± 1.01
**2-Fe**	>100	>100
**2**	3.7 ± 0.4	6.0 ± 0.7
**3-Fe**	63.5 ± 2.1	>100
**3**	40.6 ± 2.5	27.7 ± 2.9
**4-Fe**	73.5 ± 0.7	>100
**4**	88.5 ± 0.7	32.0 ± 10.4
**5-Fe**	>100	>100
**5**	7.7 ± 0.3	7.9 ± 0.5
**Cisplatin**	2.4 ± 0.4	2.2 ± 0.3

^*a*^The IC_50_ values were determined by the MTT assay after 48 h of compound exposure. Data represents the mean ± SD of at least three independent experiments made in triplicates.

**Table 2:** Cytotoxicity of 1, 2, 3, 4 and 5 against PC-3, Z-138 and JURKAT cancer cell lines and HMLE, MCF 10A, 1BR3G and CCD-18Co non malignant cells.

IC_50_ (μM) ^*a*^	Hemolysis (%)^*b*^							
	Cell line							
Compound	PC-3	Z-138	JURKAT	HMLE	MCF-10A	1BR3G	CCD-18Co	
**1**	8.5 ± 1.4	4.8 ± 1.2	15.1 ± 3.1	6.4 ± 04	18.8 ± 2.9	12.7 ± 1.6	27.0 ± 4.4	0
**2**	3.8 ± 0.2	4.4 ± 0.3	7.2 ± 1.9	7.0 ± 0.3	6.5 ± 1.3	7.5 ± 0.5	20.7 ± 0.3	0
**3**	35.0 ± 4.2	16.5 ± 1.4	>100	32.5 ± 3.8	39.7 ± 2.9	45.0 ± 1.0	>100	0
**4**	>100	23.1 ± 1.8	>100	47.7 ± 4.5	>100	78.0 ± 8.5	>100	0
**5**	7.7 ± 0.3	2.9 ± 0.4	5.4 ± 1.3	6.1 ± 0.1	23.8 ± 1.3	18.0 ± 4.2	53.5 ± 3.5	0

^*a*^ The IC_**50**_ values were determined by the MTT assay after 48 h of treatment. Data represents the mean ± SD of at least three independent experiments made in triplicates.

^***b***^ Percentage of hemolysis at 100 μM.

To gain insight into the cytotoxic potency of ligands **1**, **2** and **5,** their antiproliferative activity was evaluated in a stable breast cancer stem (CS)-like cell line (HMLER-shEcad). This cell line was originally established from triple oncogenic transformed and immortalized human mammary epithelial cells (HMLER), wherein knockdown of E-cadherin triggered an epithelial-mesenchymal transition (EMT) that resulted in cells with features characteristic of CSCs [44,48]. As expected, CS-like HMLER-shECad cells were more resistant to the well-known chemotherapy agent doxorubicin than non-CS-like HMLER isogenic control cells [48] (IC_50_ = 0.3±0.02 μmol/L vs 0.10±0.02 μmol/L, respectively, representing a ~3-fold increase in IC_50_) (Fig 2B). In contrast, the cytotoxic profile of ligands **1** and **2** remained largely unaltered in CS-like HMLER-shECad cells (IC_50_ = 5.3±0.7 μmol/L and 6.8±0.1 μmol/L, respectively) relative to HMLER cells (IC_50_ = 6.5±0.4 μmol/L and 6.6±0.3 μmol/L, respectively) (Fig 2B), indicating that these ligands induce cell death through a mechanism that cannot by repressed by the chemoresistant-CS-like phenotype. Moreover, compound **1** displayed some selective cytotoxicity towards HMLER-shECad cells. In contrast, HMLER-shECad exhibited some resistance to ligand **5-**induced cytotoxicity (IC_50 =_ 8.6±0.5 μmol/L compared with 5.1±0.1 μmol/L in HMLER cells) (Fig 2B).

**Fig 3:**
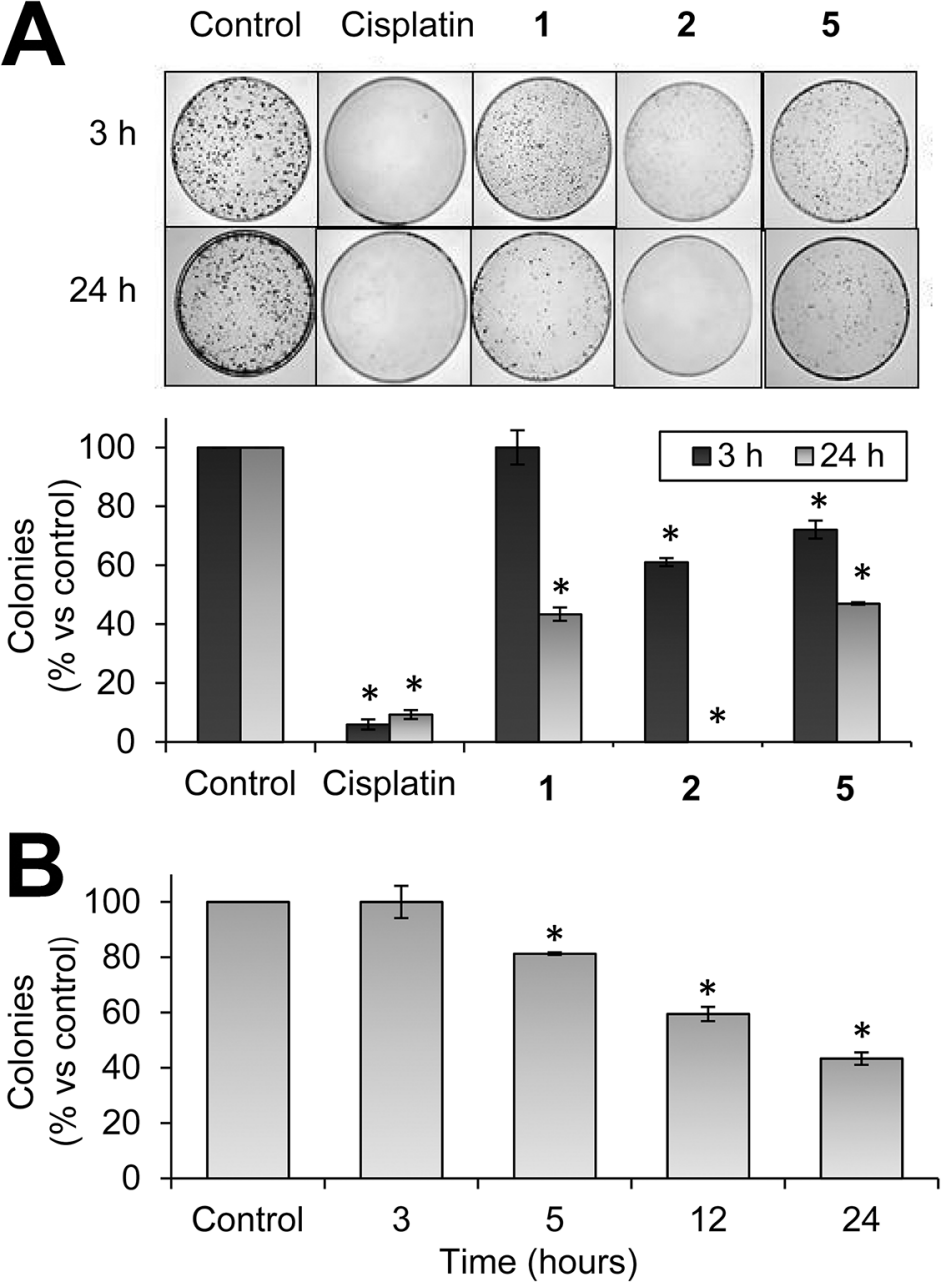
Clonogenic assay. (A) Colony formation of MCF-7 cells after exposure to compounds **1**, **2** and **5** (10 μmol/L) for 3 or 24 h. Cisplatin was included as a positive control. (B) Colony formation after exposure to compound **1** (10 μmol/L) for 3, 5, 12 and 24 h. Bar charts show the percentage of counted colonies relative to control untreated cells and represent the mean±SD of 3 independent experiments. *p < 0.05 versus control cells.

The long-term activity of the ligands was determined by measuring their ability to inhibit the clonogenic potential of cancer cells. Thus, MCF-7 cells were treated for 3 or 24 h with 10 μmol/L of ligand **1**, **2** or **5,** or cisplatin as a positive control, followed by plating at low density. Analysis of colony numbers after 10 days revealed a marked inhibitory effect of compound **2** on colony formation and the number of colonies was significantly reduced by 39% compared with control cells after 3 h exposure to the ligand (Fig 3A). Furthermore, the clonogenicity of MCF-7 cells was almost abolished after 24 h exposure to compound **2,** revealing a greater inhibitory activity than cisplatin. At this time point, compounds **1** and **5** also significantly reduced the colony numbers by 57% and 53%, respectively, although their activity was lower than compound **2**, which is in agreement with the antiproliferative activity of the ligands (**Table 2**). In contrast to cisplatin treatment, inhibition of cell growth by ligands was time-dependent. Exposure of MCF-7 cells to ligand **1** for 3, 5, 12 and 24 h reduced the number of colonies by 0%, 18.7%, 40.5% and 56.6%, respectively (Fig 3B). These results indicate that the ligands trigger a delayed cell death mechanism that requires several hours to take place.

### Compounds 1, 2 and 5 promote cell cycle arrest and apoptosis

**Fig 4:**
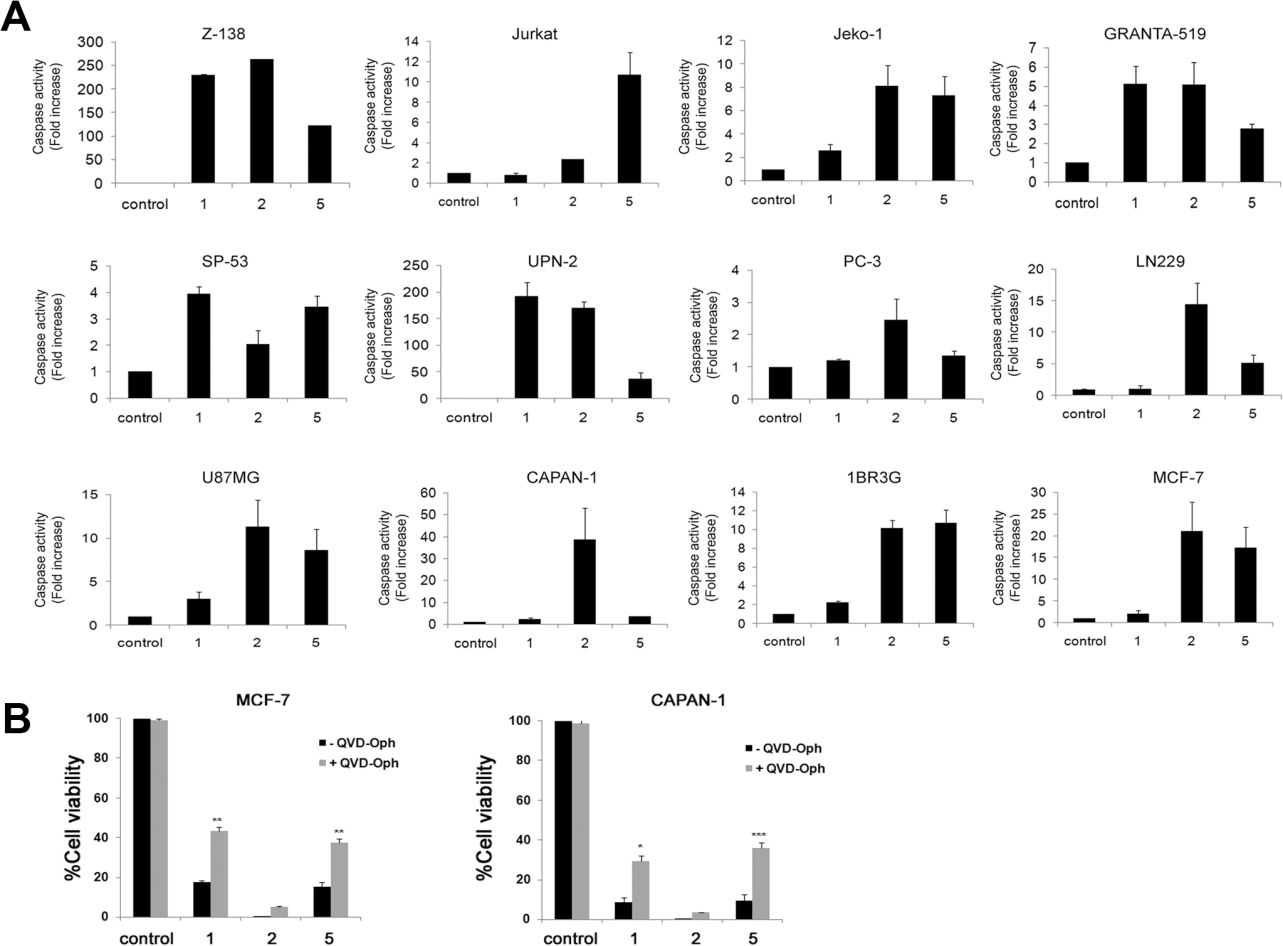
Induction of caspase activity. (A) The indicated cell lines were treated for 48 h with compounds **1**, **2** and **5** (10 μmol/L) and caspase 3/7 activity was measured as indicated in Materials and Methods. Bar charts show the fold increase in caspase activity relative to untreated (control) and represent the mean±SD from three independent experiments performed in triplicate. (B) MCF-7 and CAPAN-1 cells were treated for 48 h with the ligands (10 μM) in the absence (-) or presence (+) of the pan-caspase inhibitor QVD-Oph (20 μM) and cell viability was measured with the MTT assay. The data shows the percentage of viable cells relative to untreated cells (control). Data represents the mean ± SD of 3 independent experiments performed in triplicate. The differences between absence and presence of QVD-OPh treatment were statistically significant at * p<0.05, **p<0.01 and ***p<0.001.

To determine whether the ligands induce cellular death through the activation of programmed cell death (apoptosis), the activation of the executioner caspases, caspase-3 and -7, was analyzed using a luminometric assay in a panel of human cancer cell lines. Cells were treated with the ligands at 10 μmol/L and caspase activity was monitored after 48 h. All three ligands activated caspase 3/7 to some extent (Fig 4). Compound **2** treatment clearly increased caspase 3/7 activity in all cell lines in comparison with untreated controls. Interestingly, treatment with compound **1** led to significant caspase 3/7 activation in lymphoma (Z-138, Jeko-1, Granta and SP-53) but not in leukemia (JURKAT) or glioma (LN229 and U87MG) cell lines. Compound **5** induced a broad pro-apoptotic effect, activating caspase 3/7 in most cell lines except PC-3 cells, and was the most effective compound against JURKAT cells (Fig 4). Importantly, these results correlate strongly with the profile of cytotoxic effects induced by compounds **1**, **2** and **5** (Fig 2A), and suggest that compounds **2** and **5** promote cell death chiefly by inducing apoptosis. These results were confirmed by analyzing the effect of caspase inhibition on the cytotoxic activity of the compounds. As shown in Fig 4B, the pan-caspase inhibitor QVD-Oph significantly reverted the cytotoxicity of compounds **1** and **5** in MCF-7 and CAPAN-1 cells inducing an increase in cell viability ranging from 2.5 to 4 fold. Noteworthy, in agreement with our previous observations, compound **2** displayed a very high cytotoxic activity, which may explain the lack of reversion in the presence of the caspase inhibitor in these experimental conditions. These findings support that the cytotoxic activity of these compounds involves caspase-dependent apoptosis.

**Fig 5:**
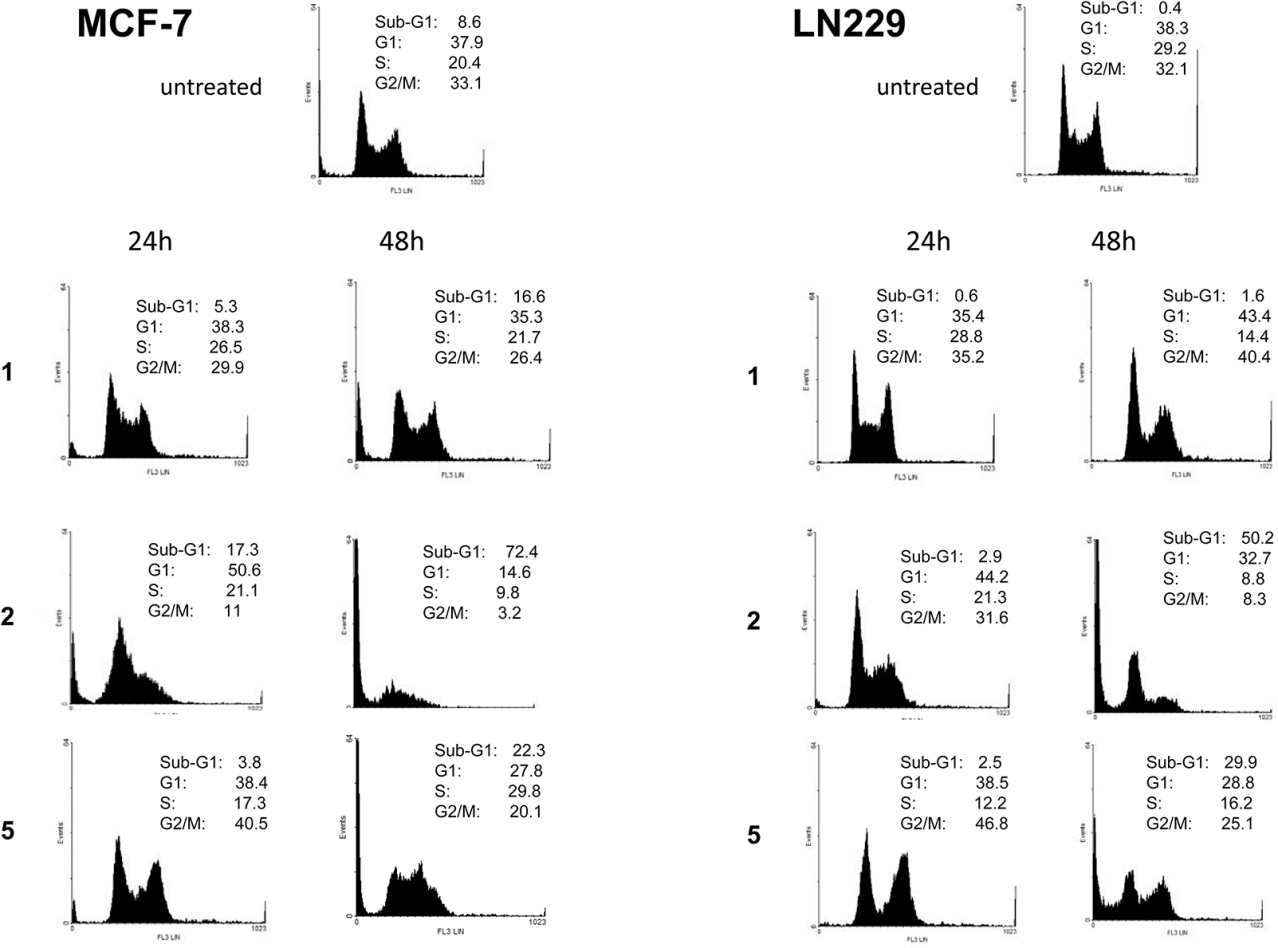
Effect of ligands on cell cycle distribution. MCF-7 cells (left) and LN229 cells (right) were left untreated (top) or treated with the indicated ligands (10 μmol/L) for 24 and 48 h. Cell cycle profiles were obtained by flow cytometry of propidium iodide-stained cells. The quantified percentage of cells in each cell cycle phase is indicated.

To explore the effect of ligands on cell cycle progression, the cell cycle distribution of MCF-7 and LN229 cells was examined by flow cytometry after 24 and 48 h exposure to compounds **1**, **2** and **5** (10 μmol/L). In agreement with its robust cytotoxic activity in both cell lines, compound **2** increased the proportion of cells in G1 at 24 h, followed by a dramatic induction of apoptosis at 48 h as indicated by the increase in the sub-G1 population (Fig 5). In contrast, compound **1** exerted only a modest effect on the cell cycle, which was apparent after 48 h as indicated by a small induction of apoptosis in MCF-7 cells and a reduction in the S-phase fraction in LN229 cells. Interestingly, in both cell lines, compound **5** treatment resulted in partial G2/M arrest at 24 h, followed by a marked induction of apoptosis at 48 h (Fig 5).

### Compounds 1, 2 and 5 are inducers of oxidative stress

**Fig 6:**
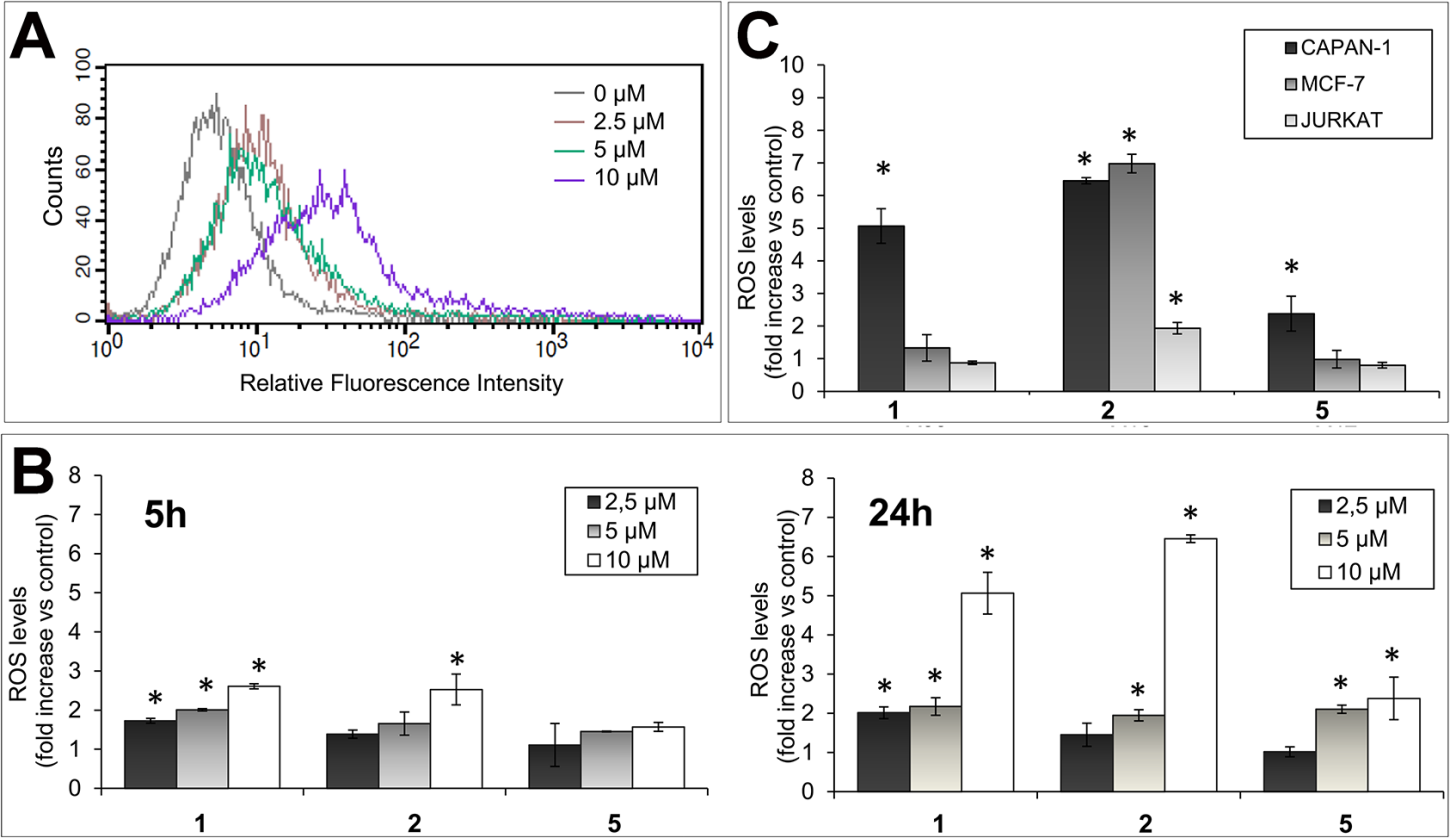
Oxidative activity of ligands. (A) CAPAN-1 cells were incubated with 0, 2.5, 5 or 10 μmol/L of compound **1** for 24 h and intracellular ROS levels were determined with H_2_DCFDA staining. Histogram shows the dose-dependent increase of the probe fluorescence intensity. (B) ROS levels (relative to untreated cells) after exposing CAPAN-1 cells to the indicated concentrations of compounds **1**, **2** or **5** for 5 or 24 h. (C) ROS levels (relative to untreated cells) after exposing CAPAN-1, MCF7 and JURKAT cells to 10 μmol/L of **1**, **2** and **5** for 24 h. Differences versus untreated control cells were considered significant at *p < 0.05. Data represents the mean±SD from 3 independent experiments.

To determine whether the compounds induce oxidative stress, ROS accumulation was evaluated in CAPAN-1 cells using the non-polar cell permeable probe H_2_DCFDA. Once inside cells, the acetate groups of the probe are enzymatically cleaved generating the nonfluorescent derivative H_2_DCF, which emits strong green fluorescence on oxidation by ROS [49]. Exposure of CAPAN-1 cells to increasing concentrations of compound **1** for 24 h resulted in a dose-dependent induction of ROS as measured by an increase in fluorescence from 5.8 (0 μmol/L) to 10.9 (2.5 μmol/L), 11.79 (5 μmol/L), and 32.6 (10 μmol/L) (Fig 6A). Exposure of CAPAN-1 cells to equal concentrations of **1**, **2** and **5** for 5 and 24 h revealed that all three ligands could generate intracellular ROS in a dose- and time-dependent manner (Fig 6B). Consequently, CAPAN-1 cells exposed to compound **1** for 5 h exhibited a 1.73, 2.01 and 2.61-fold increase of ROS levels at 2.5, 5, and 10 μmol/L, respectively. Equivalent concentrations of compound **2** resulted in a 1.39, 1.66 and 2.53-fold increase in ROS (Fig 6B). At this time point, compound **5** exhibited lower oxidative activity than **1** and **2** at all concentrations. Importantly, ROS continued to be produced and, after 24 h treatment with ligands at 10 μmol/L, the intracellular ROS levels were 6.4-fold (**2**), 5.1-fold (**1**) and 2.4 fold (**5**) higher than in untreated cells (Fig 6B), pointing to a strong oxidative activity of the ligands in this cell line.

To explore the generality of ROS production, MCF-7 and JURKAT cells were likewise exposed to 10 μmol/L of compounds **1**, **2** and **5** for 24 h. Results revealed a differential oxidative activity of the ligands in individual cell lines. In MCF-7 cells, compound **2** generated a significant 7-fold increase in ROS levels (Fig 6C), which was equivalent to the ROS induction detected in CAPAN-1 cells. The oxidative activity of compound **1** in MCF-7 cells was, however, lower than in CAPAN-1cells. In JURKAT cells, ROS levels were significantly increased only with compound **2** (1.94 folds versus control cells; Fig 6C).

**Fig 7:**
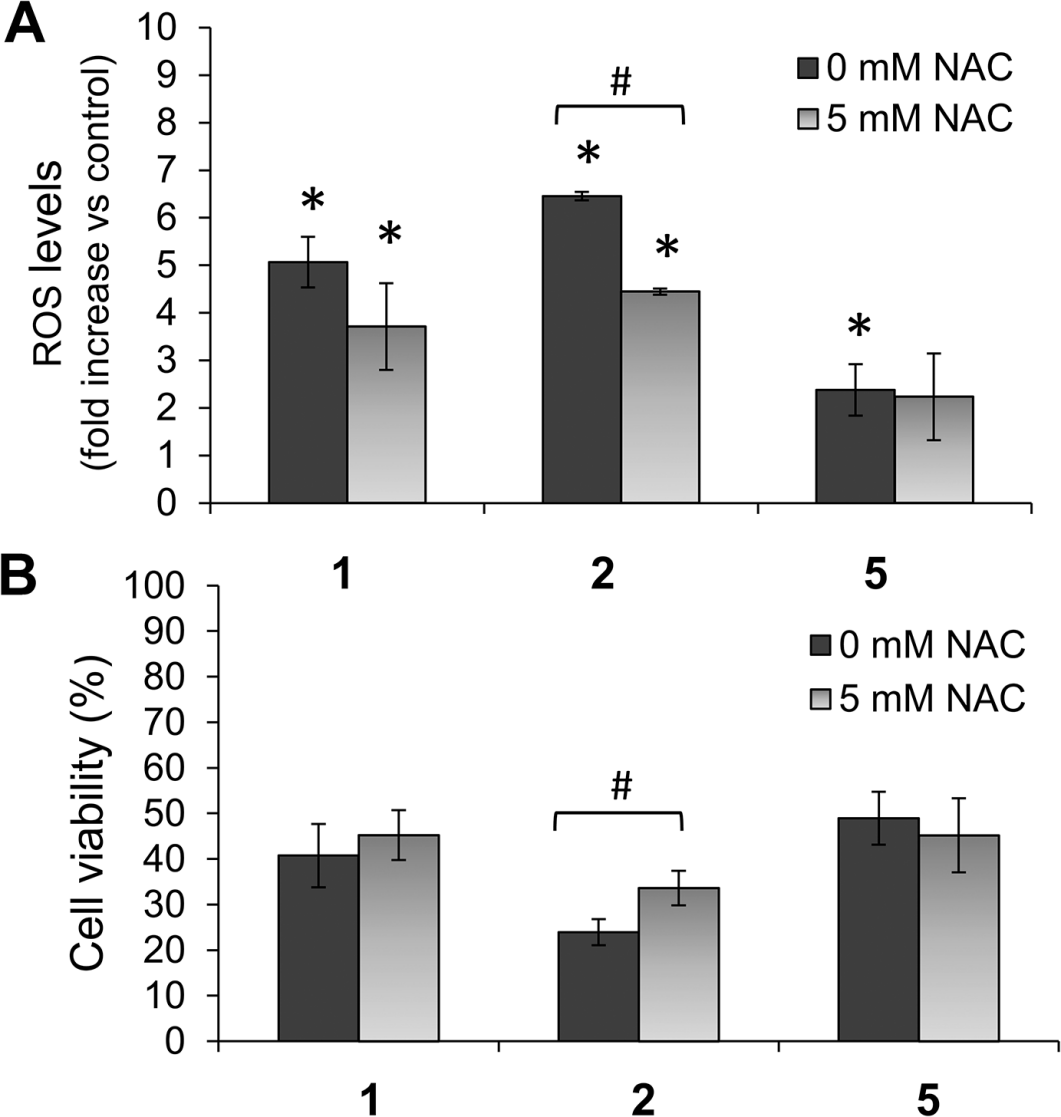
Effects of NAC on compound 1, 2 and 5 activity. CAPAN-1 cells were incubated with 10 μmol/L of **1**, **2** or **5** for 24 h with or without NAC (5 mmol/L). (A) Changes in the ROS levels were determined with H_2_DCFDA staining. (B) Changes in cell viability were determined by MTT assay. The results are presented as a percentage of untreated cells and represent mean±SD of 3 independent experiments. *p < 0.05 versus control cells. ^#^p < 0.05 versus cells without NAC treatment.

To assess the relationship between prooxidant properties and cytotoxic activity of the ligands, we studied whether the widely-used ROS scavenger N-acetylcysteine (NAC) could inhibit ligand-induced cytotoxicity. NAC treatment (5 mmol/L) reduced levels of ROS induced by compounds **1** and **2** in CAPAN-1 cells by 31.1% and 26.8%, respectively; however, the effect of NAC on the oxidative activity of **5** was more modest (Fig 7A). Furthermore, CAPAN-1 cell viability significantly increased from 23.9±2.9% when exposed to compound **2** (5 μmol/L) in the absence of NAC to 33.6±3.8% in the presence of 5 mmol/L NAC, representing a 40.6% increase in cell survival (Fig 7B). CAPAN-1 cell viability also increased from 40.7±6.9% with compound **1** (10 μmol/L) without NAC to 45.2±5.4% in the presence of NAC, while no protective effect of NAC was observed for compound **5** cytotoxicity (Fig 7B).

### Compounds 1, 2 and 5 chelate intracellular labile iron

**Fig 8:**
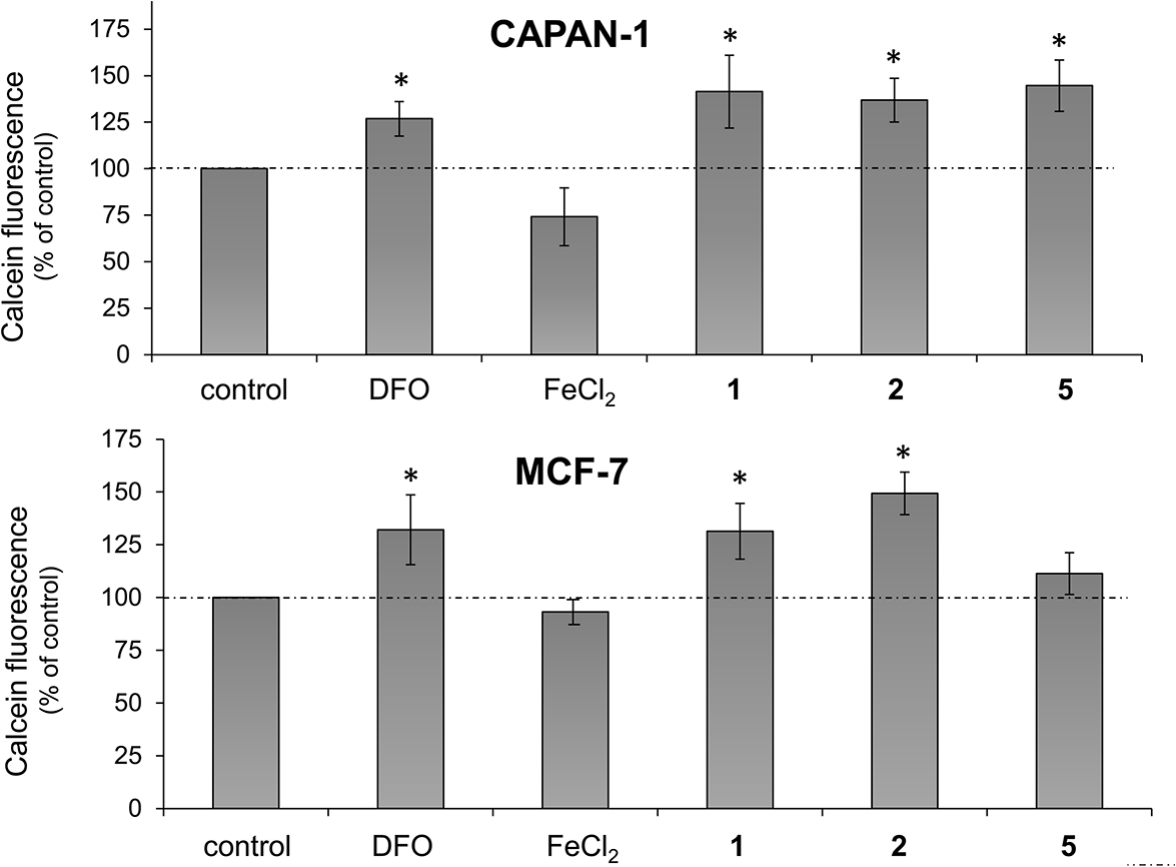
Chelating activity of 1, 2 and 5 in CAPAN-1 cells. CAPAN-1 cells were treated with 10 μmol/L of 1, 2 or 5, deferoxamine (DFO) (100 μmol/L) and FeCl_2_ (100 μmol/L) for 24 h and the intracellular labile iron was determined with calcein-AM. Data represents the mean±SD of 3 independent experiments. *p < 0.05 versus control cells.

We analyzed whether ligand-induced ROS generation was associated with their strong capacity to bind iron [38–43], forming iron coordination species inside cells. Thus, the effect of compounds **1**, **2** and **5** on the intracellular labile iron pool in CAPAN-1 and MCF-7 cells was determined using the iron-sensitive probe calcein-AM, a cell membrane-permeable molecule that is rapidly hydrolyzed in the cytosol to release the fluorescent probe calcein. Calcein fluorescence is quenched stoichiometrically upon binding to intracellular metals, mainly to labile iron [50,51]. The classic iron chelator agent deferoxamine (DFO) was used to estimate the labile iron pool in CAPAN-1 and MCF-7 cells [52]. Treatment of CAPAN-1 cells with ligands at 10 μmol/L for 24 h prior to incubation with calcein-AM significantly increased the fluorescence intensity of the probe to 141.4±19.5% (**1**), 136.8±11.7% (**2**) and 144.6±13.8% (**5**) of untreated cells (Fig 8A), revealing a decrease in the intracellular iron content. Incubation of the cells with DFO at 100 μmol/L for 24 h resulted in a similar increase in calcein fluorescence, indicating that the cellular chelatable iron was complexed by DFO to a similar extent to the ligands (Fig 8A). In contrast, exposure of CAPAN-1 cell to 100 μmol/L FeCl_2_ for 24 h led to a quenching effect on calcein that resulted in a fluorescence decrease to 74.2±15.5% of control cells (Fig 8A). Similar iron-binding capacity was detected for compounds **1** and **2** in MCF-7 cells since pre-incubation with the ligands significantly increased calcein fluorescence by 131.4±13.2% (**1**) and 133.8±10.02% (**2**) compared with untreated cells (Fig 8B), which was equivalent to the fluorescence increase observed after DFO incubation (132.1±16.5%). However, only a moderate iron-chelating effect was detected for compound **5** in MCF-7 cells (Fig 8B).

### The cytotoxicity of compounds 1, 2 and 5 is not associated to intracellular iron depletion

**Fig 9:**
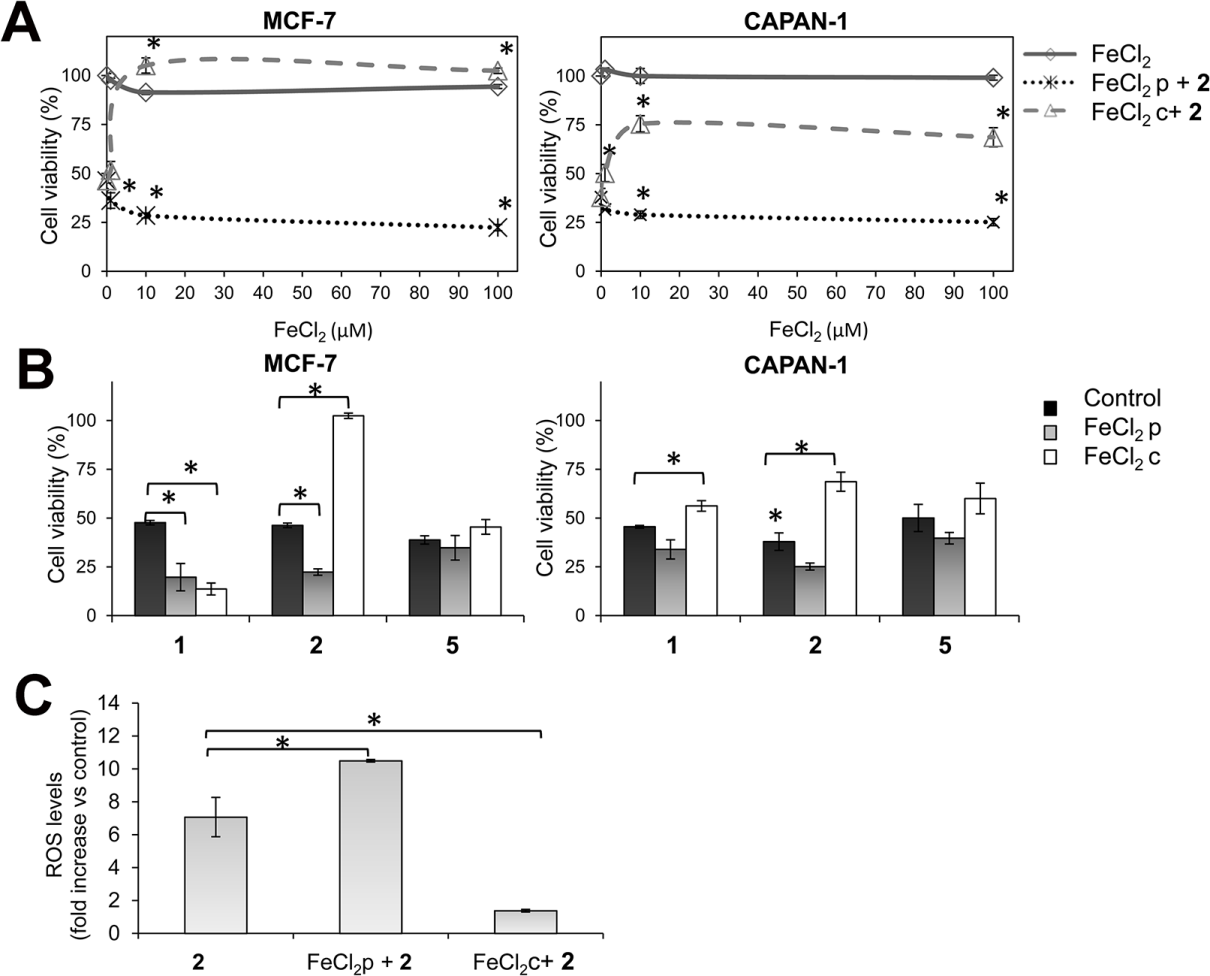
Effects of iron on 1, 2 and 5 activity. (A) Effect of 2 h FeCl_2_ pre-incubation (FeCl_2_ p) (dotted line) or co-incubation (FeCl_2_ c) (dashed line) at 1, 10 or 100 μmol/L on the cytotoxicity of **2** (5 μmol/L) in MCF-7 and CAPAN-1 cells. FeCl_2_ alone was included as a control (solid line). Cell viability was determined after 48 h of treatment by the MTT assay. (B) Effect of FeCl_2_ pre-incubation or co-incubation (100 μmol/L) on the cytotoxicity of **2** (5 μmol/L), **1** and **5** (10 μmol/L). Cells treated with the ligands alone were included as a reference (control). (C) ROS induction in CAPAN-1 cells after 24 h treatment with **2** (10 μmol/L) alone, together with FeCl_2_ or after FeCl_2_ pre-incubation (at 100 μmol/L). Results are presented as a percentage of untreated cells and represent mean±SD of 3 independent experiments. *p < 0.05 versus control cells.

Given the above, we addressed whether the depletion of intracellular iron by the chelating activity of the ligands plays a role in their cytotoxicity. Thus, the antiproliferative activity of compound **2** (5 μmol/L) in CAPAN-1 and MCF-7 cells was determined after pretreatment of cells with increasing concentrations of FeCl_2_ for 2 h in order to increase the intracellular iron content and balance the depletion of iron provoked by the ligands. Cells were also exposed to equivalent concentrations of FeCl_2_ alone to exclude any iron-induced cytotoxicity. Results showed that treatment with FeCl_2_ alone did not affect the viability of MCF-7 or CAPAN-1 cells at any tested concentration (Fig 9A). In contrast, FeCl_2_ pretreatment increased the cytotoxicity of compound **2** in an iron concentration-dependent manner (Fig 9A), resulting in a significant reduction in MCF-7 and CAPAN-1 cell viability by 51.8% and 37.7%, respectively, in cells pretreated with 100 μmol/L FeCl_2_ (Fig 9B). Remarkably, when FeCl_2_ was co-incubated with compound **2,** the cytotoxic effect of the ligand in both cell lines was clearly inhibited (Fig 9A and 9B), probably because the corresponding non-active iron-complex (**2-Fe**) was rapidly generated in the cell culture medium. FeCl_2_ pretreatment also significantly enhanced compound **1** cytotoxicity in MCF-7 and CAPAN-1 cells (by 58.6% and 25.5%, respectively), while FeCl_2_ co-incubation inhibited the cytotoxicity of the ligand in CAPAN-1, but not in MCF-7 cells (Fig 9B). These findings are in agreement with our previous results showing that the corresponding iron complex (**1-Fe**) was cytotoxic against MCF-7 cells (IC_50_ = 17.5 μmol/L) while it was not active against CAPAN-1 cells (IC_50_>100 μmol/L). Neither pretreatment nor co-incubation with FeCl_2_ affected compound **5** cytotoxicity, particularly in MCF-7 cells, which may be explained by the reduced oxidative activity of this ligand in this cell line (Fig 9B).

The enhanced cytotoxicity of compound **2** in FeCl_2_-pretreated CAPAN-1 cells was associated with a significant increase in its oxidative activity, resulting in a 1.5-fold increase in ROS levels compared with non-pretreated cells. In contrast, extracellular complexation of compound **2** to iron by FeCl_2_ co-incubation abolished ROS induction (Fig 9C).

### Compounds 1, 2 and 5 induce oxidative DNA damage

**Fig 10:**
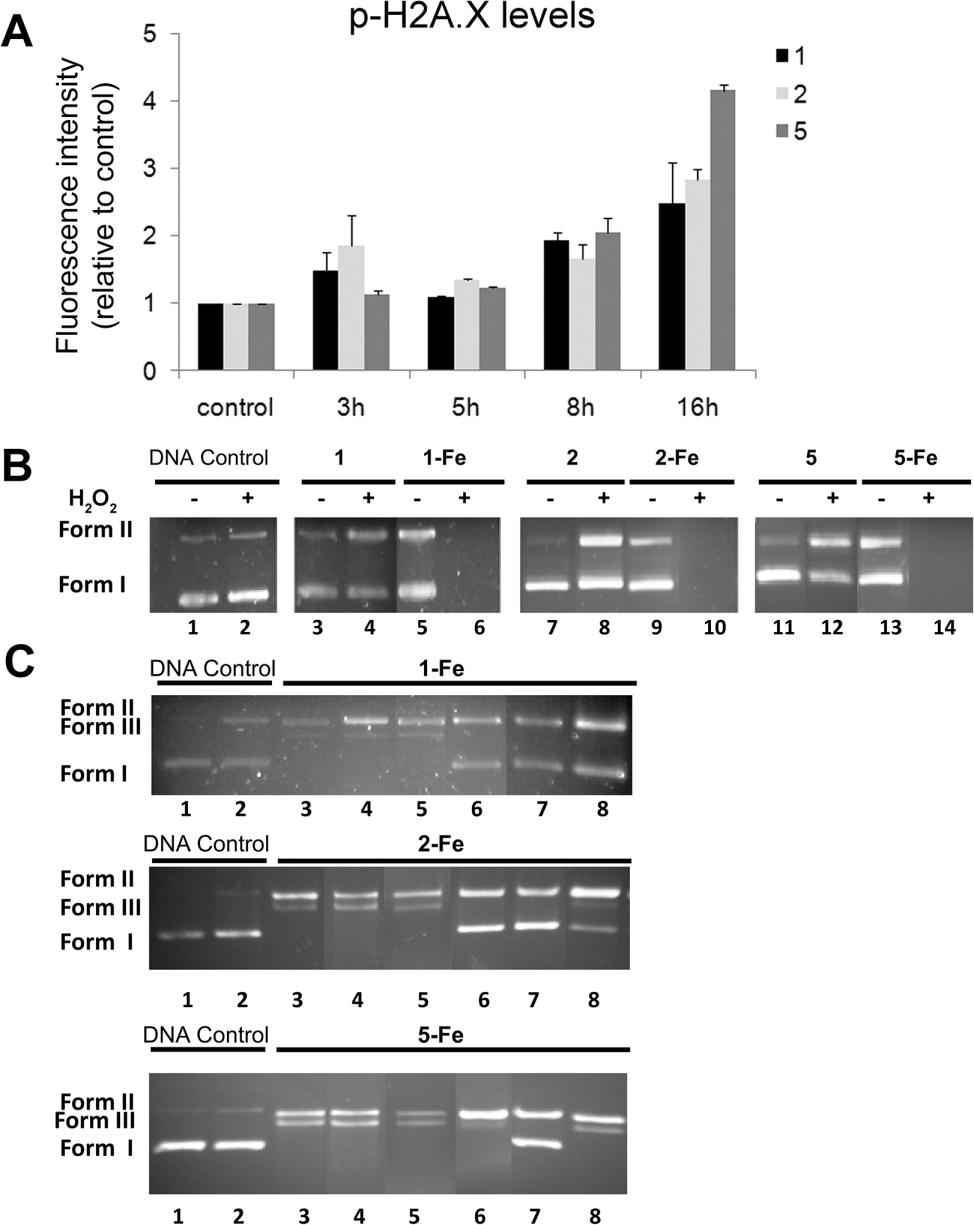
Analysis of the DNA damage induced by 1, 2 and 5. (A) Quantification of cellular DNA damage. MCF-7 cells were exposed to 10 μmol/L **1**, **2** and **5** for the indicated times and the levels of p-H2A.X were quantified using flow cytometry after immunostaining with anti-pH2A.X specific antibodies. The graph represents the mean fluorescence intensity for each experimental condition, relative to untreated cells. (B) Electrophoretic analysis of DNA cleavage. Supercoiled pUC18 plasmid DNA (18.9 μmol/L) was incubated with ligands **1, 2** and **5** and their respective Fe(II) complexes **1-Fe, 2-Fe** and **5-Fe** at 25 μM for 1 h (37°C). Lane 1, DNA control; lane 2, DNA control + H_2_O_2_; lane 3, 5, 7, 9, 11, 13, ligand or Fe(II) complex in the absence of H_2_O_2_; lane 4, 6, 8, 10, 12, 14, ligand or Fe(II) complex in the presence of H_2_O_2_. (C) Effect of groove binders and ROS scavengers on the cleavage of supercoiled pUC18 plasmid DNA treated with complexes **1-Fe, 2-Fe** and **5-Fe** at 15 μmol/L for 1 h (37°C) in the presence of H_2_O_2_. Lane 1, DNA control; lane 2, DNA control + H_2_O_2_; lane 3, complex control in the absence of potential inhibitors; lane 4, Hoechst 40 μmol/L; lane 5, methyl green 20 μmol/L; lane 6, Tiron 10 mmol/L; lane 7, sodium azide 0.4 mol/L; lane 8, 3 μL of DMSO.

The ability of compounds **1**, **2** and **5** to induce DNA damage was evaluated at the cellular level by analyzing the phosphorylation of histone H2A.X on serine 139, a well-established cellular marker of DNA double-stranded breaks [53]. Exposure to ligands (10 μmol/L) resulted in a time-dependent increase in phosphorylated H2A.X in MCF-7 cells, between three fold (**1** and **2**) and four fold (**5**) (Fig 10A).

To complete the analysis, we evaluated the capacity of the ligands to directly interact with DNA using supercoiled pUC18 DNA and gel electrophoresis. The nuclease activity of ligands **1, 2** and **5** (25 μmol/L) and their respective iron complexes **1-Fe, 2-Fe** and **5-Fe** (25 μmol/L) was measured as the extent of the conversion of supercoiled DNA (Form I) to open circular DNA (Form II) and/or linear DNA (Form III) in the presence and absence of hydrogen peroxide. As expected, **1-Fe**, **2-Fe** and **5-Fe** displayed strong nuclease activity in the presence of hydrogen peroxide, leading to a complete degradation of plasmid DNA under the conditions established for the assay (Fig 9B lanes 6, 10 and 14, respectively; Table 3). When the concentration was decreased to 15 μmol/L, **1-Fe**, **2-Fe** and **5-Fe** were able to induce total conversion of the supercoiled DNA (Form I) to a nicked circular form (Form II) and linear form (Form III) (Fig 10C lane 3) by double strand breaks in the plasmid DNA. The relative proportions of the different forms of plasmid DNA after the treatments are detailed in Table 4. The DNA cleavage activity of the different compounds was studied in the presence of Hoechst (a minor DNA groove blocker) [54,55] and methyl green (a major DNA groove blocker) [56,57]. As shown in Fig 10C (lines 4 and 5) and Table 4, the amount of linear DNA was not reduced by addition of specific DNA groove blockers, indicating that the nuclease activity takes place without any groove selectivity. The involvement of ROS in the nuclease mechanism was confirmed by monitoring the inhibition of DNA cleavage in the presence of ROS scavengers (Fig 10C lines 6,7 and 8; Table 4) [58]. Accordingly, the addition of tiron (a superoxide radical scavenger), sodium azide (a singlet oxygen scavenger) and dimethylsulfoxide (a hydroxyl radical scavenger), reduced DNA cleavage activity of **1-Fe** and **2-Fe**, indicating the involvement of different ROS in the DNA cleavage reactions. In contrast, **5-Fe** activity was reverted only by addition of sodium azide [59], suggesting that the nuclease activity of this complex is likely to be associated with singlet oxygen generation.

**Table 3:** Relative proportions of different forms of plasmid DNA after 1, 1-Fe, 2, 2-Fe, 5 and 5-Fe treatments.

	Proportion [%]	
Form*:	I	II
**Untreated**	86.9	13.1
**H**_**2**_ **O**_**2**_	83.7	16.3
**1**	84.3	15.7
**1 + H**_**2**_ **O**_**2**_	52.8	47.2
**1-Fe**	61.8	38.2
**1-Fe + H**_**2**_ **O**_**2**_	0	0
**2**	91.3	8.7
**2 + H**_**2**_ **O**_**2**_	56.8	43.2
**2-Fe**	74.1	25.9
**2-Fe + H**_**2**_ **O**_**2**_	0	0
**5**	92.3	7.7
**5 + H**_**2**_ **O**_**2**_	53.3	46.7
**5-Fe**	64.8	35.2
**5-Fe + H**_**2**_ **O**_**2**_	0	0

* I: supercoiled form, II: open form.

**Table 4:** Relative proportions of different forms of plasmid DNA after 1-Fe, 2-Fe and 5-Fe treatments in the presence of groove binders and ROS scavengers.

	Proportion [%]								
	1-Fe			2-Fe			5-Fe		
Form*:	I	II	III	I	II	III	I	II	III
**Untreated**	89.2	10.8	0	99.8	0.2	0	98.1	1.9	0
**H**_**2**_ **O**_**2**_	59.3	40.7	0	95.5	4.5	0	94.7	5.3	0
**Complex + H**_**2**_ **O**_**2**_	0	82.4	17.6	0	84.0	16.0	0	56.9	43.1
**Complex + H**_**2**_ **O**_**2**_ **+ Hoescht**	0	86.8	13.2	0	63.7	36.3	0	52.8	47.2
**Complex + H**_**2**_ **O**_**2**_ **+ Methyl green**	0	90.7	9.3	0	78.6	21.4	0	51.0	49.0
**Complex + H**_**2**_ **O**_**2**_ **+ Tiron**	47.6	52.4	0	60.7	39.3	0	0	96.7	3.3
**Complex + H**_**2**_ **O**_**2**_ **+ Sodium Azide**	41.8	58.2	0	63.6	36.4	0	54.5	45.5	0
**Complex + H**_**2**_ **O**_**2**_ **+ DMSO**	57.5	42.5	0	26.0	62,7	11.3	0	74.8	25.2

* I: supercoiled form, II: open form, III: linear form.

## Discussion

Exploiting the differences between normal and cancer cells is an essential step to develop innovative cancer therapies. In this regard, the distinction between redox setpoints in these two cell types represents a valuable therapeutic window that might permit redox-targeting interventions to potently and selectively eliminate cancer cells with constitutively upregulated levels of ROS [5]. Theoretically, a pro-oxidant deviation that might be well tolerated by nonmalignant cells could rapidly reach a cell-death threshold in malignant cells already at a high setpoint of constitutive oxidative stress. This hypothetical scenario prompted us to study the suitability of five highly oxidant iron complexes with selected aminopyridine ligands (**1-Fe**, **2-Fe, 3-Fe, 4-Fe** and **5-Fe)**, which were expected to be potent ROS inductors together with the corresponding uncomplexed ligands, as potential antitumoral agents. Our results demonstrate that the iron complexes failed to display any relevant cytotoxic activity, whereas the iron-free organic counterparts were cytotoxic. In particular, compounds **1**, **2** and **5** exhibited strong antiproliferative activity against a broad panel of molecularly diverse human cancer cells with IC_50_ values in the low micromolar range. Importantly, the cytotoxic activity profile of compounds **1** and **2** remained unaltered in EMT-induced stable populations of cancer stem-like cells, which characteristically exhibit resistance to the majority of commonly employed anti-cancer agents including the well-known ROS inducer doxorubicin [60,61].

The apparently counterintuitive cytotoxicity of the aminopyridine ligands can be explained from the studies of cellular Fe(II) chelation, which show that Fe(II) from the labile iron pool is efficiently chelated by the metal-free ligands. Thus, it appears reasonable to propose that the inactivity of the synthesized iron complexes must be related to the impossibility of charged species to cross the cell membrane since iron conjugation confers a positive charge to the apolar nature of the ligands, leading to less lipid-soluble molecules with impaired ability to cross the cell membrane [62]. Conversely, neutral organic ligands can readily traverse the cell membrane and form the highly oxidizing iron complexes in situ [26–28, 30–31].

Ligands **1** and **2** were found to be strong inducers of oxidative stress, leading to a greater than 5-fold increase in ROS levels in CAPAN-1 cells. The oxidative activity of compound **5** was rather more modest. Interestingly, the kinetic profile of ROS accumulation paralleled the results obtained in clonogenic assays, indicating that prolonged exposure to ligands is required to exceed the threshold levels of oxidative damage capable of compromising cell growth. ROS accumulation strongly correlated with the induction of oxidative DNA damage and preceded the activation of caspases and the onset of apoptosis, indicating that the ligands promote delayed cell death through oxidative mechanisms. Indeed, results obtained with NAC demonstrated that ROS reduction enhanced the cellular survival to compounds **1** and **2** treatments, confirming that oxidative stress is, in part, responsible for cell death. Nevertheless, NAC had little effect on the cytotoxicity of compound **5,** suggesting that alternative mechanisms may be involved in its cytotoxicity, in agreement with its unique ability to promote G2/M arrest.

Remarkably, a lower oxidative activity of the ligands was detected in MCF-7 and JURKAT cells compared with CAPAN-1 cells, despite similar cytotoxicity. The vulnerability of cancer cells to oxidative stress is greatly dependent on the particular pathways dysregulated in the cells as well as on their antioxidative capacities [6,63]. Consequently, different alterations in ROS levels may lead to similar cytotoxic outcomes in different tumors. For instance, chronic lymphocytic leukemia lymphocytes are reported to have a predominant oxidative stress status, which may favor an enhanced cytotoxic response to prooxidant interventions [64,65]. Nonetheless, it cannot be ruled out that other mechanisms may be contributing to the cytotoxic activity of the ligands, particularly for compound **5**.

We examined whether the depletion of intracellular iron by the chelating activity of the ligands may be involved in their antitumor activity. Iron is essential for cell growth and DNA synthesis, and iron deprivation can lead to cellular death [66,67]. Different studies and clinical trials have demonstrated that iron-chelators are effective anti-cancer agents [68–70]. Our results showed that iron overload with FeCl_2_ salts failed to reverse the cytotoxic activity of the ligand in cells. On the contrary, higher intracellular iron levels led to increased cytotoxicity of the compounds presumably because the intracellular formation of the oxidizing iron-complexes was enhanced, resulting in a significant increase in the amount of ROS. Hence, the antitumor effect of the ligands relies on their strong oxidative activity rather than their iron-chelating capacity. These experiments also confirmed that the extracellular generation of iron-complexes by co-incubation of the ligands with FeCl_2_ salts clearly inhibits their cytotoxic activity.

Different anticancer compounds with metal binding properties can induce DNA strand breaks when binding redox active metals in the presence of oxygen [71]. The bleomycin family of glycopeptide antibiotics constitutes paradigmatic examples with utility in current chemotherapy. It is well established that bleomycin cytotoxicity is founded on a metal-dependent prooxidant mechanism that leads to DNA fragmentation. Bleomycin binds ferrous iron and O_2_ and after one-electron reduction in vivo produces an activated intermediate, a ferric hydroperoxide species [BLM-Fe(III)-OOH], which cleaves DNA by hydrogen abstraction [5]. Similarly, ligands **1**, **2** and **5** demonstrated DNA cleavage activity in cells, with kinetics that mirrored intracellular ROS accumulation. Analysis of the interaction of ligands with naked DNA revealed that only the Fe-complexed ligands displayed nuclease activity by inducing double strand breaks in the DNA in the presence of hydrogen peroxide. The DNA cleavage activity was quenched by different ROS scavengers, revealing that the nuclease activity of **1-Fe** and **2-Fe** involve different ROS, while the activity of **5-Fe** is likely to be associated to singlet oxygen generation. Further, the nuclease activity took place without any DNA groove selectivity. Collectively, these results indicate that once bound to intracellular iron, the ligands induce a strong oxidative DNA damage through ROS, which results in double-stranded DNA breaks.

**Fig 11:**
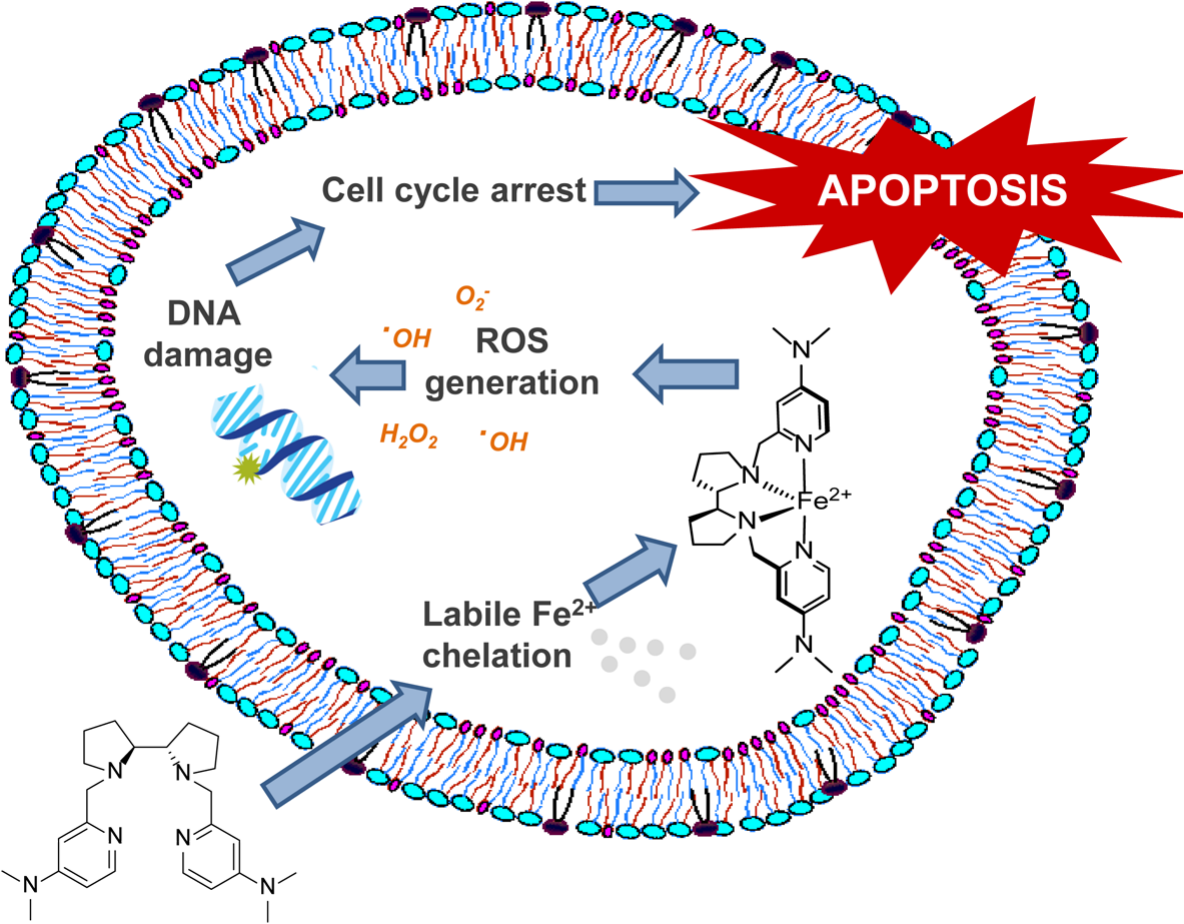
Mode of action of the amine-pyridine ligands. The cytotoxic activity of amine-pyridine ligands involves different inter-dependent processes: intracellular Fe(II) chelation, generation of ROS, DNA fragmentation through oxidative mechanisms, induction of cell cycle arrest and apoptosis.

The anti-cancer activity of amine-pyridine-based iron complexes relies on different inter-dependent processes, involving intracellular Fe(II) chelation, generation of ROS, DNA fragmentation through oxidative mechanisms, induction of cell cycle arrest and apoptosis (Fig 11). This mode of action is clearly associated with the observed cytotoxic effects of **1** and **2**. Additional mechanisms may be involved in the anticancer activity of **5**, which displays similar antiproliferative and proapoptotic activities but limited generation of ROS.

Cancer cells have increased steady-state ROS levels and are likely to be more vulnerable to damage by further ROS insults induced by exogenous agents [1,72]. Indeed, the cell-killing activity of the vast majority of currently used anti-cancer therapies is mostly related to a commonly shared ability, directly or indirectly, to generate ROS [69]. Accordingly, drug resistance phenotypes, including those of multidrug resistant tumor- and metastasis-initiating CS-like cellular states, can be explained in terms of resistance to ROS-induced apoptotic killing. In a call for a much faster timetable for developing new curative anti-cancer strategies, it has been recently proposed that greater efforts must be made on “oxidative therapy” as a strategy against the current incurability of metastatic cancers [7]. Although it is acknowledged that future studies will have to confirm any beneficial *in vivo* effects and the nature of interaction as cocktail partners either with current ROS-generating radio- and chemo-therapeutic regimens or with the newer therapies that do not directly generate ROS, our current findings illustrate that, upon chelation of intracellular iron, the pro-oxidant activity of amine-pyrimidine-based iron complexes efficiently kills cancer and ROS-refractory cancer stem-like cells. Thus, our study provides functional evidence for promising redox-directed anti-cancer metallodrugs.
